# Rottlerin Reduces cAMP/CREB-Mediated Melanogenesis via Regulation of Autophagy

**DOI:** 10.3390/ijms20092081

**Published:** 2019-04-27

**Authors:** Nurinanda Prisky Qomaladewi, Mi-Yeon Kim, Jae Youl Cho

**Affiliations:** 1 Department of Integrative Biotechnology, Sungkyunkwan University, Suwon 16419, Korea; prisky95@skku.edu; 2 School of Systems Biomedical Science, Soongsil University, Seoul 06978, Korea

**Keywords:** melanogenesis, autophagy, rottlerin, cAMP/CREB signaling pathway

## Abstract

Melanogenesis is the sequential process of melanin production by melanocytes in order to protect the skin from harmful stimuli. Melanogenesis is disrupted by radiation exposure, which results in the differentiation of melanocytes into melanoma. Recently, some methods have been developed to maintain the instability of melanogenesis in melanoma by activating cellular autophagy. However, there is still a lack of knowledge about how autophagy is involved in the regulation of melanogenesis in melanoma cells. Here, we used rottlerin as an autophagy inducer to investigate the role of the cyclic adenosine monophosphate (cAMP)/cAMP response element binding (CREB) signaling pathway in melanogenesis. We found that rottlerin can inhibit melanin production by targeting cAMP, which is initially activated by alpha-melanocyte stimulating hormone (α-MSH). Our findings suggest that rottlerin has a pivotal role as an autophagy inducer in the regulation of melanogenesis by targeting the cAMP/CREB signaling pathway.

## 1. Introduction

Skin is the largest organ of the body and is divided into three layers—comprised of different cell types—the epidermis, dermis, and hypodermis. The skin functions to protect and maintain homeostasis in our body. One type of cell in the epidermis produces and stores a dark pigment (melanin) and is called a melanocyte [1]. Melanogenesis is the complex processes, by which melanin is produced [2]. In general, the function of melanin production is to protect the skin from harmful stimuli such as UV rays, visible light, and infrared irradiation, by absorbing it and transferring it to keratinocytes, which are surrounded by melanocytes, resulting in cell pigmentation and eventually, protection against skin cancers [3,4]. However, abnormalities in melanin production and distribution can result in hyperpigmentation by overregulating some dedicated cellular pathways involved in melanogenesis, and transformation of melanocytes into malignant melanoma cells [5]. Therefore, many methods to control the regulation of melanogenesis have been studied.

Recently, the relationship between melanogenesis and autophagy has become an interesting target to regulate melanogenesis. Autophagy, known as a survival pathway with respect to starvation, has a crucial role in the regulation of melanosome biogenesis by the activation of MITF (microphthalmia-associated transcription factor) and melanosome removal [6]. Unfortunately, the relationship between autophagy and melanogenesis regulation is not well understood.

**Figure 1:**
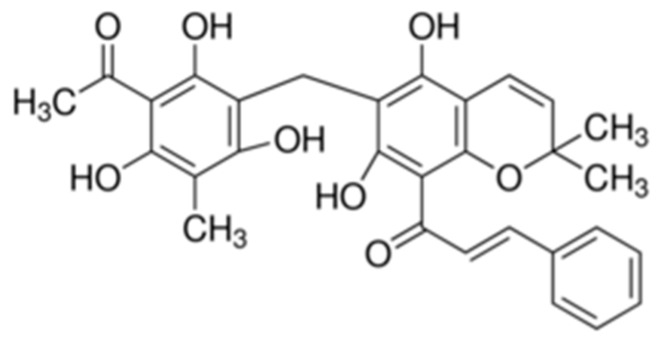
Chemical structure of rottlerin.

Rottlerin (Figure 1) is a polyphenol compound abundant in a tropical plant found in India, Australia, the Philippines, and some countries in Southeast Asia [7,8]. It is a known inducer of autophagy and cell death by inhibiting the action of mTORC1 (mammalian target of rapamycin complex 1) through the AMPK/mTOR (adenosine monophosphate-activated protein kinase/mammalian target of rapamycin) cascade [9]. Rottlerin plays various roles in the response of some cancer cells to autophagy [10,11].

In this study, we examined how rottlerin—as an autophagy inducer—takes part in regulating melanogenesis, particularly through the cyclic adenosine monophosphate (cAMP)/cAMP response element binding (CREB) signaling pathway in melanoma cells. This study will provide further insight into the relationship between autophagy and melanogenesis in melanoma.

## 2. Results

### 2.1. Effects of Rottlerin on Melanin Produced by Melanoma Cells

**Figure 2:**
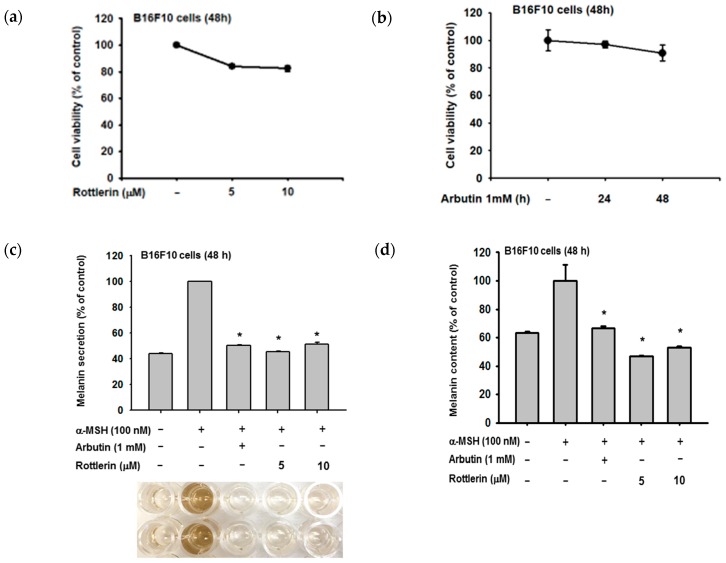
Effect of rottlerin on alpha-melanocyte stimulating hormone (α-MSH)-induced melanogenesis in melanoma cells. (**a**) and (**b**) Viability of B16-F10 cells after rottlerin treatment (5 and 10 µM) and arbutin (1 mM at 48 h) was assessed with MTT solution. (**c**) Extracellular and (**d**) intracellular melanin contents in B16-F10 cells (10^5^ cells/mL) induced with α-MSH (100 nM), treated with rottlerin (5 and 10 µM) or arbutin (1 mM) as a positive control for 48 h were determined by spectrophotometry. All data (**a**–**c**) are expressed as the mean ± the standard deviation (SD) of three replicates. * *p* < 0.05 compared to control or normal groups.

We examined the relationship between rottlerin concentrations and melanin production by B16-F10 melanoma cells, based on extracellular and intracellular melanin levels (Figure 2b,c, respectively). Rottlerin concentrations of 5 and 10 µM inhibited melanin production when compared to the arbutin positive control, without affecting cell viability (Figure 2a,b).

### 2.2. Effects of Rottlerin on the Expression of Some Genes Related to Melanogenesis

**Figure 3:**
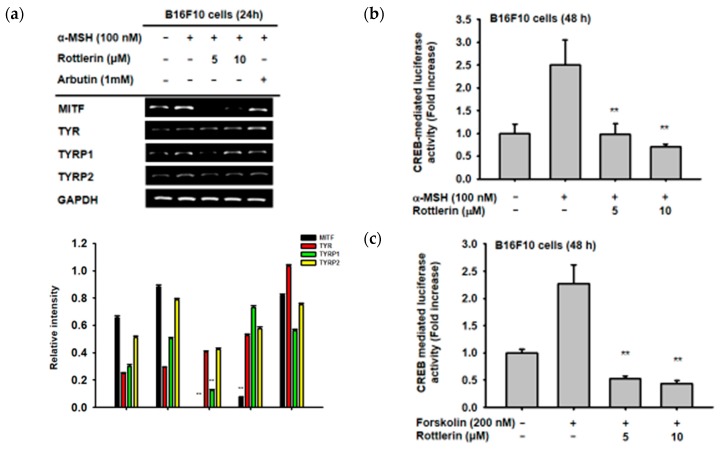
Effect of rottlerin on genes involved in melanogenesis and CREB (cAMP response element binding) transcription factor. (**a**) Semi-quantitative PCR was carried out to measure mRNA expression of MITF, TYR (tyrosinase), TYRP1 (tyrosinase related protein 1), and TYRP2 in B16-F10 cells (10^5^ cells/mL) stimulated by α-MSH in the presence or absence of 5 and 10 µM of rottlerin. (**b**) and (**c**) B16-F10 cells (10^5^ cells/mL) were transfected with CREB-luciferase (CREB-Luc) and beta-galactosidase (β-gal, 0.8 µg) for 48 h, activated with α-MSH 24 h after CREB-Luc transfection, and (**c**) treated with forskolin (200 nM) with or without 5 and 10 µM of rottlerin. All data (**b**,**c**) are expressed as the mean ± SD of three replicates. ** *p* < 0.01 compared to control groups.

In order to investigate which genes, related to melanogenesis, are affected by the presence of rottlerin, B16-F10 cells were treated with α-MSH to induce the expression of mRNA from some genes related to melanogenesis, as reported previously [12,13,14]. Treatment with rottlerin at concentrations of 5 and 10 µM significantly decreased the level of *MITF* and *TYRP1* (Tyrosinase related protein 1) mRNA expression, but not that of tyrosinase (*TYR*) or *TYRP2* (Figure 3a). These results imply that rottlerin could inhibit melanogenesis by reducing the expression of some genes related to melanogenesis.

### 2.3. Effects of Rottlerin on CREB Transcription Factor Activation

Since rottlerin can reduce the gene expression of *MITF*, we performed a luciferase reporter assay to investigate the relationship between MITF and the CREB signaling pathway. As shown in Figure 3b,c, both 5 and 10 µM of rottlerin significantly decreased CREB-mediated luciferase activity induced by α-MSH and forskolin, respectively, which indicated that rottlerin inhibited melanogenesis through the cAMP/CREB signaling pathway.

### 2.4. Effect of Rottlerin on the Melanogenesis-Related Signaling Pathway

**Figure 4:**
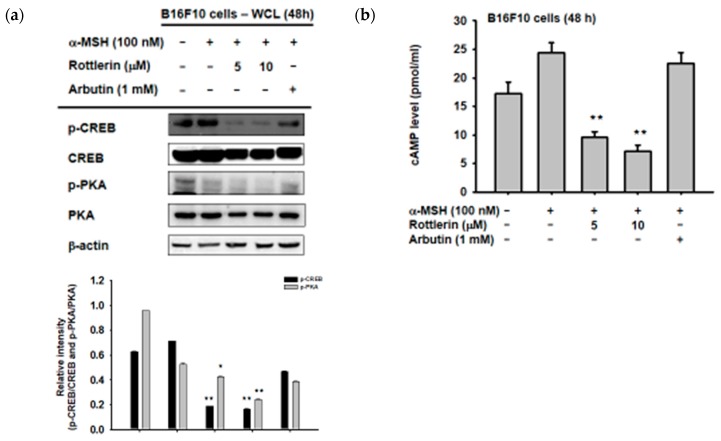
Rottlerin inhibits melanogenesis via the cAMP/CREB signaling pathway. (**a**) B16-F10 cells (10^5^ cells/mL) were activated by α-MSH in the presence or absence of rottlerin (5 and 10 µM) or arbutin (1 mM) for 48 h. Phosphorylated and total CREB were evaluated by immunoblotting. (**b**) Intracellular cAMP levels were analyzed using a cAMP immunoassay kit. All data (**b**) are expressed as the mean ± SD of three replicates. WCL: Whole cell lysates. * *p* < 0.05 and ** *p* < 0.01 compared to control groups.

We first examined the phosphorylation activity of CREB in the cytosol by immunoblotting, to explore the exact mechanism by which rottlerin inhibits melanogenesis in B16-F10 melanoma cells through the cAMP/CREB signaling pathway. As expected, Figure 4a shows that the phosphorylation of CREB was downregulated by the presence of rottlerin at concentrations of 5 and 10 µM, after induction by α-MSH, as well as the upstream of CREB, which is PKA (protein kinase A). Moreover, the cAMP level in the cells was also reduced by treatment with rottlerin after induction with 100 nM of α-MSH (Figure 4b), which implied that rottlerin inhibited melanogenesis in melanoma cells by targeting cAMP/CREB signaling and reducing the process of melanin synthesis.

### 2.5. Effects of Rottlerin on the cAMP/CREB Signaling Pathway by Regulation of Autophagy

**Figure 5:**
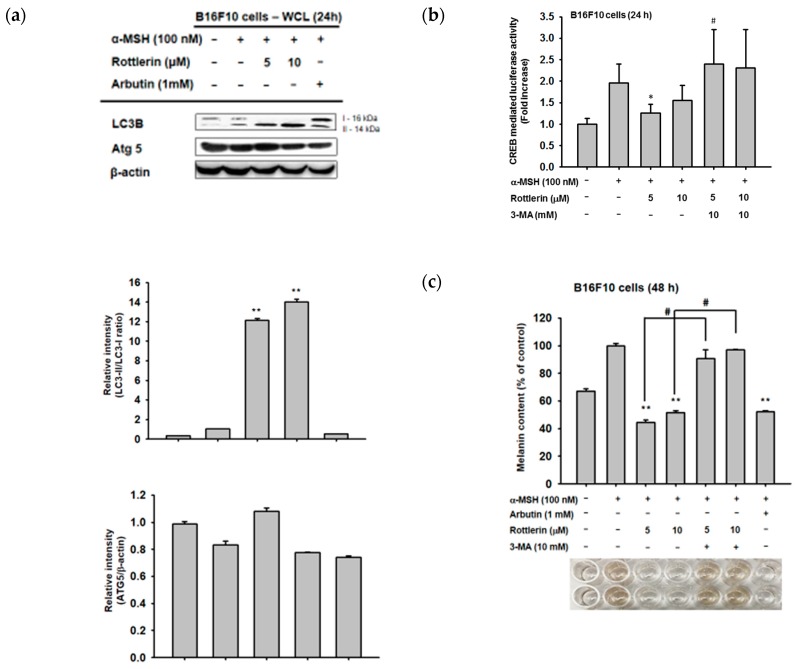
Rottlerin downregulates CREB-mediated melanogenesis by the activity of autophagy. (**a**) Confirmation of rottlerin as an autophagy inducer was achieved by assessing levels of autophagy-related proteins, such as LC3 and ATG5, by immunoblotting analysis under the same conditions for 24 h. (**b**) B16-F10 cells (10^5^ cells/mL) were transfected with CREB-Luc and β-gal (0.8 µg) for 24 h, activated with α-MSH 24 h after CREB-Luc transfection, and (**c**) treated with α-MSH with or without 5 and 10 µM of rottlerin and 3-MA (10 mM). Luciferase activity was measured with a luminometer. (**c**) B16-F10 cells (10^5^ cells/mL) were induced by α-MSH in the presence or absence of rottlerin (5 and 10 µM) or arbutin (1 mM) as well as 3-MA (10 mM) for 48 h, and level of intracellular melanin content was measured by spectrophotometry. All data (**b**,**c**) are expressed as the mean ± SD of three replicates. * *p*< 0.05 and ** *p* < 0.01 compared to control groups. # *p* < 0.01 compared to selected groups.

Since rottlerin is a well-known autophagy inducer in some cancer cells, we examined whether the capacity of rottlerin to downregulate the cAMP/CREB pathway is due to the activation of autophagy by rottlerin. We confirmed that rottlerin activates autophagy in B16-F10 melanoma cells by the upregulation of LC3B-II level in the cytosol (Figure 5a). Moreover, we treated rottlerin concomitantly with one well-known autophagy inhibitor, 3-MA, to understand whether this compound attenuates rottlerin-mediated anti-melanogenesis activity. As shown in Figure 5b,c, 3-MA recovered the luciferase activity mediated by CREB and reduced the inhibitory level of the intracellular melanin content under rottlerin exposure in α-MSH-treated B16-F10 cells, which implied that rottlerin may target the cAMP/CREB signaling pathway and positively regulate melanogenesis by the regulation of autophagy.

## 3. Discussion

In this study, we aimed to investigate the role of rottlerin as an autophagy inducer in the regulation of melanogenesis in melanoma cells. We observed how rottlerin-mediated autophagy modulated melanogenesis through the cAMP/CREB pathway by targeting cAMP. Melanogenesis is the process by which melanocytes produce melanin in order to protect the skin from harmful radiation, and it occurs through several stages and involves several kinds of gene regulation pathways. One of the major factors involved in this process is MITF, a transcription factor responsible for transcription of some genes related to melanin synthesis in the melanosome, such as tyrosinase, *TYRP1*, and *TYRP2* [15]. Activation of MITF in the nucleus arises from one major receptor that determines skin pigment phenotype, the melanocortin 1 receptor (MC1R), via interaction with the most important hormone secreted from keratinocytes to induce melanogenesis, α-MSH. This interaction next activates signaling cascades by initiating cAMP production, leading to upregulation of CREB phosphorylation and activation of MITF transcription in the nucleus [16,17].

In our study, we focused on the cAMP/CREB signaling pathway, which mediated melanogenesis via induction of α-MSH to activate the cAMP/CREB cascade in B16-F10 murine melanoma cells. We found that autophagy, represented by rottlerin as an autophagy inducer, decreased extracellular and intracellular melanin content by inhibiting the production of melanin by B16-F10 melanoma cells after induction by α-MSH. This finding suggests that rottlerin inhibits melanin production through this cascade. Moreover, we found that rottlerin reduced the activity of CREB as a transcription factor in the nucleus, subsequently decreasing the mRNA level of some genes related to melanogenesis, such as *MITF* and *TYRP1*. These results support our hypothesis regarding autophagy’s role in regulating melanogenesis, specifically through the cAMP/CREB pathway.

We determined the specific cellular protein inhibited by rottlerin, by examining the proteins in the pathway, including CREB phosphorylation and the amount of cAMP in the cells. Since cAMP is upstream of CREB, and our results showed that cAMP level was significantly decreased after rottlerin treatment compared to arbutin treatment (a well-known commercially available skin whitening agent), we concluded that rottlerin inhibited melanogenesis in melanoma cells by reducing cAMP production. 

Rottlerin is a known regulator of autophagy and apoptosis [18]. It has several actions through autophagy-related cascades, thereby regulating several diseases, including inflammation and various cancers [7,10,19]. Thus, in the human melanoma cell line SK-Mel 28, the presence of protective autophagy in the cell was found to cause the death of the cells by inhibition of mTORC1, resulting in protein loss [20]. This inhibition turned on autophagy-inducing signaling by the lipidation of LC3 to form autophagosome [21]. We confirmed that rottlerin could upregulate the expression of LC3 cellular protein in melanoma cells, which is one of the crucial proteins responsible for the formation of the autophagosome without involving the autophagy-related protein (ATG) system [21,22,23]. Moreover, we also found rottlerin-mediated suppression of cAMP/CREB-mediated luciferase activity and that the melanogenesis process is managed by autophagy activity (Figure 5b,c). Namely, co-treatment with 3-MA, an autophagy inhibitor displaying suppression of PI3K/AKT to inhibit mTORC1 [24], strongly abrogated both CREB-mediated luciferase activity and the α-MSH-induced melanogenesis process (Figure 5b,c), demonstrating that the process of melanogenesis was able to be regulated by autophagy. 

**Figure 6:**
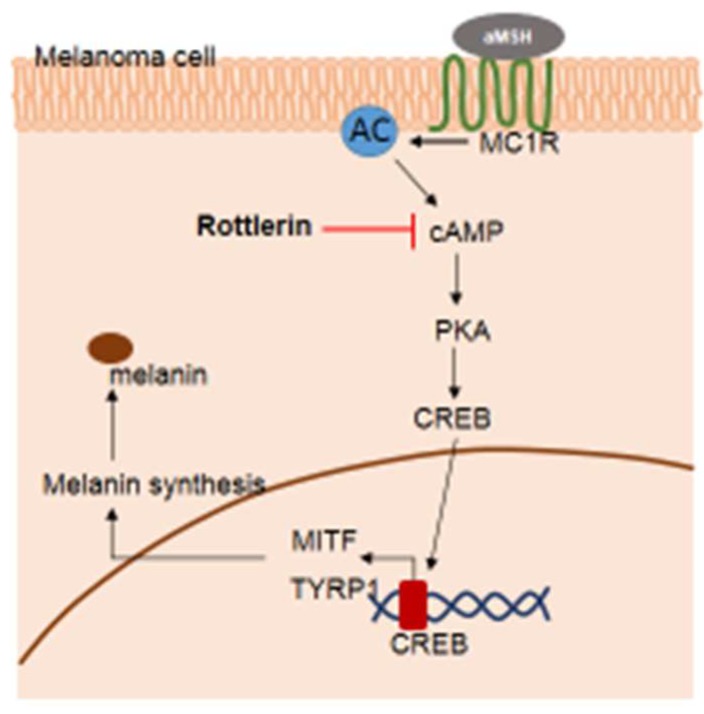
Potential pathway by which rottlerin regulates melanogenesis in melanoma cells.

In conclusion, rottlerin—as an autophagy inducer—played a role in inhibiting melanogenesis by targeting cAMP in the cytosol through the cAMP/CREB signaling pathway (Figure 6). Future studies examining the exact stage of autophagy, which inhibits melanogenesis in melanoma cells, are necessary in order to understand the relationship between autophagy and melanogenesis in melanoma. The findings may support autophagy as a target in skin cancer therapy.

## 4. Materials and Methods

### 4.1. Materials 

Rottlerin was purchased from Calbiochem (San Diego, CA, USA). B16-F10 cells are a murine melanoma cell line from a C57BL/6J mouse (No. CCL-6475) from ATCC (Rockville, MD, USA). The cell culture products used were: TRIzol, fetal bovine serum (FBS), penicillin/streptomycin, DMEM as well as high modified DMEM were purchased from Gibco Products (Grand Island, NY, USA). Phosphate-buffered saline (PBS) was from Capricorn Scientific (Ebsdorfergrund, Germany). The luciferase constructs with cAMP response element binding (CREB) binding promoter sites were used as previously reported in Reference [25]. The polyethyleneimine (PEI), alpha-melanocyte-stimulating hormone (α-MSH), arbutin, tetrazole 3-(4,5-dimethylthiazol-2-yl)-2,5-diphenyltetrazolium bromide (MTT), sodium dodecyl sulfate (SDS), sodium hydroxide (NaOH), dimethyl sulfoxide (DMSO), and 3-methyladenine (3-MA) were obtained from Sigma Chemical Co. (St. Louis, MO, USA). The RT-PCR primers were from Bioneer (Seoul, Korea). The phospho-specific and total antibodies against: LC3B, ATG5, and PKA were from Cell Signaling Technology (Beverly, MA, USA); CREB was from Abcam (Cambridge, UK); and β-actin was from Santa Cruz Biotechnology (Santa Cruz, CA, USA).

### 4.2. Drug Preparation, Cell Culture, and Cell Viability Assay

A stock solution of rottlerin was prepared by dilution to a concentration of 10 mM using DMSO. The B16-F10 melanoma cells were cultured in DMEM media supplemented with 1% penicillin/streptomycin and 10% FBS. The cells were incubated at 37 °C and 5% CO_2_. To determine cell cytotoxicity of the drug, the B16-F10 cells (1 × 10^5^ cells/mL) were pre-incubated for 18 h and then treated with rottlerin (5 and 10 µM). The cytotoxic effects of rottlerin were evaluated by the MTT assay as previously reported [26].

### 4.3. Determination of Extracellular and Intracellular Melanin Content

The B16-F10 cells (1 × 10^5^ cells/mL) were plated in 12-well plates and incubated overnight at 37 °C and 5% CO_2_. The seeded cells were treated with or without rottlerin, α-MSH, 3-MA, and arbutin for 48 h using high-modified DMEM (10% FBS plus 1% penicillin/streptomycin) media. To determine the extracellular melanin content, the media was removed after 48 h of incubation, transferred to 96-well plates, and the optical density was measured under 405 nm. The media was then removed and cells were washed with PBS, lysed with lysis buffer—containing 20 mM Tris-HCl pH 7.4, 2 mM ethyleneglycotetraacetic acid, 50 mM β-glycerophosphate, 1 mM sodium orthovanadate, 1 mM dithiothreitol, 1% Triton X-100, 10% glycerol, 10 µg/mL aprotinin, 10 µg/mL pepstatin, 1 mM benzamide and 2 mM PMSF—and incubated for 10 min before centrifugation. The supernatants were used as whole cell lysates for immunoblotting and pellets containing intracellular melanin were solubilized in 1 M NaOH with 10% DMSO at 42 °C to be measured at 405 nm with an optical density reader.

### 4.4. Preparation of Cell Lysate for Immunoblotting

The supernatants, containing protein from the melanin content, were analyzed by immunoblotting as previously reported [27,28]. The total and phosphorylated levels of LC3B (14,16), ATG5 (56), CREB (phospho-form 46; total form 40), PKA (42) and β-actin (45) were visualized with an ECL system (Amersham, Little Chalfont, Buckinghamshire, UK).

### 4.5. Measurement of mRNA Levels by Reverse Transcription-Polymerase Chain Reaction

To determine levels of expression of mRNA responsible for melanogenesis, B16-F10 cells were treated with rottlerin (5 and 10 µM) and arbutin 1 mM as a control, with and without α-MSH (100 nM) for 48 h. Total RNA was precipitated using TRIzol Reagent (Gibco RBL) according to the manufacturer’s instructions, and was kept at −70 °C until use. Semi-quantitative RT-PCR was conducted as previously reported. The primers (Bioneer, Seoul, Korea) used are listed in Table 1.

### 4.6. Luciferase Reporter Gene Assay

The B16-F10 cells (1 × 10^5^ cells/mL) were cultured for 18 h in a 24-well plate prior to transfection with plasmids (0.8 µg/mL each well) encoding a luciferase gene under a CREB promoter by the PEI method. After 24 h of stabilization, the transfected cells were treated with rottlerin concomitantly with α-MSH and forskolin, respectively, for the next 24 h. The luciferase activity was assessed using the Luciferase Assay System (Promega, Madison, WI, USA) as previously reported [29].

### 4.7. cAMP Assay

The cAMP level was determined using a cAMP immunoassay kit (Cayman Chemical Company, Ann Arbor, MI, USA) as previously reported [30]. The B16-F10 cells were lysed in 0.1 M HCl to inhibit the phosphodiesterase activity and incubated at room temperature for 30 min. Supernatants were collected in the e-tube. The lysates were added to a 96-well plate coated with rabbit IgG polyclonal antibody and incubated concomitantly with a constant concentration of cAMP-acetylcholinesterase (Ache) conjugate (Tracer) as a competitor of cAMP in the well. After incubation at 4 °C for 18 h, the wells were washed to remove the unbound cAMP and Ellman’s reagent was added to determine the activity of cAMP. The optical density was read at 420 nm, as proportional to the amount of the cAMP Tracer, yet inversely proportional to the concentration of cAMP in the wells.

### 4.8. Statistical Analysis

All data are presented as the mean ± standard deviation (SD) calculated from three samples. For statistical comparison, all values were analyzed using ANOVA/Scheffe’s post hoc test as well as the Kruskal–Wallis/Mann–Whitney test. Significance was reached when *p*-values were <0.05 or <0.01. The statistical analyses were performed using SPSS software (Version 24, SPSS Inc., Chicago, IL, USA). 

## Figures and Tables

**Table 1:** Sequences of primers (mouse) used in semi-quantitative RT-PCR.

Name	Primer	Sequence (5′ to 3′)
MITF	Forward	AACTCATGCGTGAGCAGATG
	Reverse	TACCTGGTGCCTCTGAGCTT
TYR	Forward	GTCCACTCACAGGGATAGCAG
	Reverse	AGAGTCTCTGTTATGGCCGA
TYRP1	Forward	ATGGAACGGGAGGACAAACC
	Reverse	TCCTGACCTGGCCATTGAAC
TYRP2	Forward	CAGTTTCCCCGAGTCTGCAT
	Reverse	GTCTAAGGCGCCCAAGAACT
GAPDH	Forward	ACCACAGTCCATGCCATCAC
	Reverse	CCACCACCCTGTTGCTGTAG

## Abbreviations

UV	Ultraviolet
MITF	Microphthalmia-associated transcription factor
PCR	Polymerase chain reaction
mTOR	Mammalian target of rapamycin
mTORC1	Mammalian target of rapamycin complex 1
α-MSH	Alpha-melanocyte stimulating hormone
AMPK	AMP-activated protein kinase
TYR	Tyrosinase
TYRP1	Tyrosinase related protein 1
TYRP2	Tyrosinase related protein 2
cAMP	Cyclic adenosine monophosphate
CREB	cAMP response element binding
3-MA	3-methyladenine
